# Effects of Alzheimer’s genetic risk scores and CSF biomarkers in de novo Parkinson’s Disease

**DOI:** 10.1038/s41531-022-00317-8

**Published:** 2022-05-11

**Authors:** Young-gun Lee, Seong Ho Jeong, Mincheol Park, Sung Woo Kang, Kyoungwon Baik, Seun Jeon, Phil Hyu Lee, Young Ho Sohn, Byoung Seok Ye

**Affiliations:** 1 Department of Neurology, Yonsei University College of Medicine, Seoul, South Korea; 2 Department of Neurology, Sanggye Paik Hospital, Inje University College of Medicine, Seoul, South Korea; 3 Brain Research Institute, Yonsei University College of Medicine, Seoul, South Korea

**Keywords:** Diagnostic markers, Medical genetics

## Abstract

Coexisting Alzheimer’s disease (AD) pathology is common in Parkinson’s disease (PD). However, the implications of genetic risk scores (GRS) for AD have not been elucidated in PD. In 413 de novo PD and 195 healthy controls from the Parkinson’s Progression Marker Initiative database, the effects of GRS for AD (GRS-AD) and PD (GRS-PD) on the risk of PD and longitudinal CSF biomarkers and clinical outcomes were explored. Higher GRS-PD and lower baseline CSF α-synuclein were associated with an increased risk of PD. In the PD group, GRS-AD was correlated positively with CSF p-tau/Aβ and negatively with CSF α-synuclein. Higher GRS-PD was associated with faster CSF p-tau/Aβ increase, and GRS-AD and GRS-PD were interactively associated with CSF α-synuclein. In the PD group, higher GRS-AD was associated with poor visuospatial function, and baseline CSF p-tau/Aβ was associated with faster cognitive decline. Higher GRS-PD was associated with better semantic fluency and frontal-related cognition and motor function given the same levels of CSF biomarkers and dopamine transporter uptake. Taken together, our results suggest that higher GRS-AD and CSF p-tau/Aβ, reflecting AD-related pathophysiology, may be associated with cognitive decline in PD patients.

## Introduction

Coexisting Alzheimer’s disease (AD) pathology is observed in approximately 30% of autopsy-confirmed Parkinson’s disease (PD) patients^1^, and mixed AD pathology is associated with more α-synuclein (αSyn)-positive Lewy body disease (LBD) pathology^2^ and faster cognitive decline^3,4^. Accordingly, multiple studies have focused on predicting AD pathology using cerebrospinal fluid (CSF) biomarkers, such as phosphorylated-tau, total-tau, and β-amyloid (Aβ), or apolipoprotein E ε4 allele (*APOE4*) in PD^5–7^. However, the contribution of CSF biomarkers on clinical progression in PD patients is conflicting. Discrepancies could originate from concomitant AD pathology^2,3^, which leads to neuronal damage and release of both neuronal tau and αSyn into CSF^8,9^ given the same level of αSyn pathology^2^. Furthermore, the status of CSF biomarkers is evolving over time^10^. Research has shown that de novo PD patients have lower CSF αSyn levels^6,7^ and that CSF αSyn decreases longitudinally in PD patients^11,12^. However, there are controversies regarding the implication of CSF αSyn in PD patients, particularly whether lower CSF αSyn is associated with motor severity^6^ and cognition^7^, or not^11,12^. Therefore, simultaneous evaluation of CSF AD biomarkers and CSF αSyn is needed to determine their exact relationships with neurodegeneration in PD patients.

Genetic risk score (GRS), based on disease-associated risk loci, captures the polygenicity of diseases and identifies an individual’s disease risk^13^. Higher GRS for PD (GRS-PD) is associated with earlier symptom onset^14,15^ and faster progression^16,17^ in PD patients. However, other studies have reported protective or no association between higher GRS-PD and the progression of PD^15,18^. As the clinical symptoms and prognosis of PD are affected by concomitant AD pathology^3^ and since there is genetic pleiotropy between AD and synucleinopathy^19^, a model that consider both GRS and CSF biomarkers of AD and PD is required. Our previous study on the Alzheimer’s Disease Neuroimaging Initiative (ADNI) dataset showed that one such approach successfully revealed correlations between GRS and CSF biomarkers for AD and PD^20^.

In this study, we computed GRS for AD (GRS-AD) and GRS-PD from the Parkinson’s Progression Marker Initiative (PPMI) database and evaluated the effects of GRS-AD and GRS-PD on longitudinal CSF biomarkers and clinical outcomes in de novo PD patients. We hypothesized that GRS-AD would predict CSF biomarkers of AD and αSyn pathologies, and that GRS-AD and CSF biomarker of AD would be related to clinical outcomes in PD patients.

## Results

### Demographic and clinical characteristics

There were no significant differences in baseline age, sex, and follow-up duration between the PD and healthy control (HC) groups. The HC group had higher education than the PD group (Table 1). GRS-PD was significantly higher in the PD group, while the distribution of *APOE4* and GRS-AD were comparable between the two groups. Compared to the HC group, the PD group had lower mean baseline CSF Aβ, total tau (t-tau), phosphorylated tau (p-tau), and αSyn levels. Baseline CSF p-tau/Aβ and amyloid positivity did not differ between the two groups. The PD group had worse motor severity scores, lower dopamine transporter (DAT) availability, and worse cognitive scores in all tests.

**Table 1:** Baseline demographics and clinical characteristics of the participants.

Variables		HCs (*N* = 195)	PD (*N* = 413)	*P*
Demographic	Age, years	60.84 (11.25)	61.65 (9.68)	0.386
	Sex, female	69 (35.4%)	143 (34.6%)	0.854
	Education, years	16.05 (2.90)	15.52 (2.97)	0.036
	Duration, months	NA	6.65 (6.55)	
	Age at onset, years	NA	59.67 (9.94)	
	Follow-up duration, years	4.41 (1.35)	4.36 (1.34)	0.675
Genetic	*APOE4* allele	0 = 145 (74.3%)	0 = 310 (75.1%)	0.98
		1 = 45 (23.1%)	1 = 93 (22.5%)	
		2 = 5 (2.6%)	2 = 10 (2.4%)	
	GRS-AD	0.20 (0.69)	0.22 (0.66)	0.675
	GRS-PD	−0.18 (0.67)	0.24 (0.73)	<0.001
CSF	Baseline Aβ_42_ (ng/mL)	1.02 (0.50)	0.90 (0.41)	0.007
		Missing data = 8	Missing data = 12	
	Baseline t-tau (pg/mL)	191.58 (79.47)	169.38 (56.96)	0.001
		Missing data = 9	Missing data = 19	
	Baseline p-tau (pg/mL)	17.52 (8.37)	14.85 (5.27)	<0.001
		Missing data = 20	Missing data = 43	
	Baseline αSyn (ng/mL)	1.70 (0.75)	1.50 (0.67)	0.003
		Missing data = 6	Missing data = 8	
	Baseline amyloid-positivity	59 (31.6%)	102 (25.4%)	0.540
		Missing data = 8	Missing data = 12	
	Baseline p-tau/Aβ_42_ ratio	19.51 (17.70)	17.56 (9.48)	0.176
		Missing data = 22	Missing data = 48	
	Longitudinal CSF measurement	166 (85.1)	354 (85.7)	0.946
Motor	UPDRS III score	1.21 (2.20)	20.79 (8.82)	<0.001
DAT scan	Mean caudate	2.98 (0.63)	2.00 (0.56)	<0.001
	Mean putamen	2.14 (0.56)	0.83 (0.30)	<0.001
	Mean striatum	2.56 (0.57)	1.41 (0.40)	<0.001
		Missing data = 3	Missing data = 3	
Cognitive	Visuospatial	13.13 (1.99)	12.77 (2.14)	0.042
	Memory	9.30 (2.32)	8.36 (2.53)	<0.001
	Semantic fluency	51.79 (11.23)	48.71 (11.67)	0.002
	Frontal/executive	46.83 (10.53)	41.12 (9.71)	<0.001
		Missing data = 0	Missing data = 1	

*Aβ*_*42*_ 42-residue amyloid-beta, *αSyn* Alpha-synuclein, *AD* Alzheimer’s disease, *APOE4* Apolipoprotein E ε4 allele, *CSF* Cerebrospinal fluid, *DAT* Dopamine transporter, *GRS* Genetic risk score, *HC* Healthy controls, *PD* Parkinson’s disease, *p-tau* Phosphorylated tau, *t-tau* Total tau, *UPDRS* Unified Parkinson’s Disease Rating Scale.

Data are expressed as a mean (standard deviation) or number (%). Results from t-tests or chi-square tests were used as appropriate.

### Factors associated with the risk of PD

**Fig. 1:**
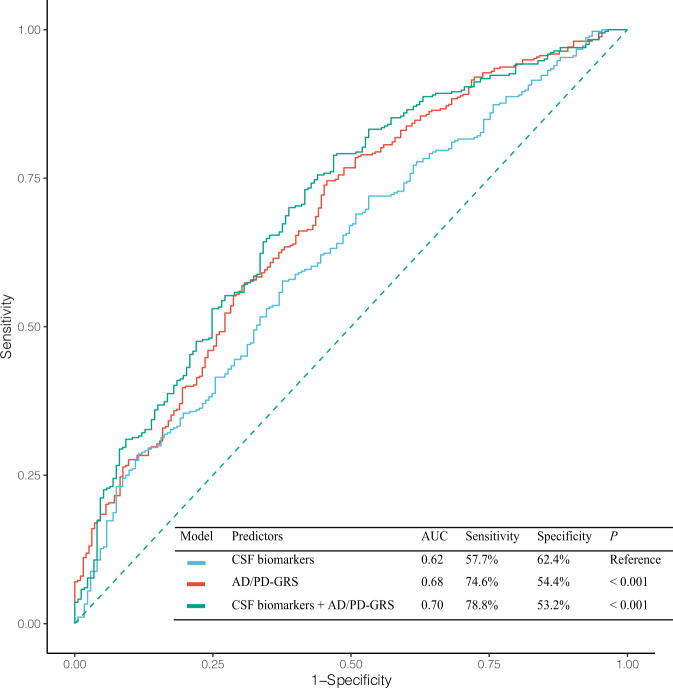
Receiver operative characteristic curve analyses for prediction of PD. Data represent the results of receiver operative characteristic curve analyses for the diagnosis of PD using CSF biomarkers, GRS for AD and PD, or both as predictors. AD Alzheimer’s disease, AUC Area under the curve, CSF Cerebrospinal fluid, GRS Genetic risk score, and PD Parkinson’s disease.

Logistic regression analyses performed in all participants showed that higher GRS-PD (model 1) and lower baseline CSF αSyn (model 2) were associated with an increased risk of PD (Table 2). Model 3 analysis showed that higher GRS-PD and lower baseline CSF αSyn were independently associated with an increased risk of PD, while GRS-AD and CSF p-tau/Aβ were not. Receiver operating characteristic (ROC) curve analyses showed that prediction models using both GRS and CSF biomarkers (AUC = 0.70, sensitivity = 78.8%, and specificity = 53.2%) or GRS only (AUC = 0.68, sensitivity = 74.6%, and specificity = 54.4%) had significantly higher diagnostic accuracy than a model using CSF biomarkers only (AUC = 0.62, sensitivity = 57.7%, and specificity = 62.4%) (Fig. 1).

**Table 2:** Effect of CSF biomarkers and genetic risk scores on the diagnosis of PD.

	Model 1		Model 2		Model 3	
	OR (95% CI)	*P*	OR (95% CI)	*P*	OR (95% CI)	*P*
Age	1.01 (0.99–1.02)	0.132	1.01 (0.99–1.02)	0.194	1.01 (0.99–1.02)	0.052
Sex, Male	1.03 (0.72–1.48)	0.560	1.06 (0.72–1.54)	0.949	1.06 (0.72–1.54)	0.736
Education	0.94 (0.89–0.99)	**0.016**	0.94 (0.88–0.99)	**0.041**	0.94 (0.88–0.99)	**0.027**
GRS-AD	1.06 (0.82–1.37)	0.683			1.00 (0.76–1.30)	0.906
GRS-PD	2.36 (1.80–3.09)	**<0.001**			2.34 (1.75–3.12)	**<0.001**
Baseline αSyn			0.69 (0.53–0.89)	**0.004**	0.69 (0.53–0.89)	**0.011**
Baseline p-tau/Aβ_42_			0.99 (0.98–1.01)	0.245	0.99 (0.98–1.01)	0.229

*αSyn* α-synuclein, *Aβ*_*42*_ 42-residue amyloid-beta, *AD* Alzheimer’s disease, *CSF* Cerebrospinal fluid, *GRS* Genetic risk score, *PD* Parkinson’s disease.

Data are presented as the results of a logistic regression model for the diagnosis of PD as a dependent variable using either GRS-AD, GRS-PD (model 1), CSF biomarkers (model 2), or both (model 3) as predictors in all subjects including healthy controls and the PD group. The covariates included age, sex, education, and the first four principal components.

Significant *P* values are indicated in bold.

### Association between GRS and CSF biomarkers

In PD patients, a linear mixed model (LMM) for time-varying CSF p-tau/Aβ showed that GRS-AD, time, and GRS-PD*time were associated with time-varying CSF p-tau/Aβ, while GRS-PD, GRS-AD*time, and time-varying CSF αSyn were not (Table 3). These results suggested that CSF p-tau/Aβ increased over time, that higher GRS-AD was associated with higher CSF p-tau/Aβ, and that higher GRS-PD was associated with a faster increase in CSF p-tau/Aβ. LMM for time-varying CSF αSyn showed that the effects of GRS-AD, time, GRS-AD*GRS-PD, GRS-AD*time, GRS-AD*GRS-PD*time, and time-varying CSF p-tau/Aβ were significant, while those of GRS-PD and GRS-PD*time were not. These results suggested that CSF αSyn decreased over time; that higher GRS-AD was associated with lower CSF αSyn, but with a slower decline in CSF αSyn; and that GRS-AD and GRS-PD interactively associated with higher CSF αSyn, but with a faster decline in CSF αSyn.

**Table 3:** Association between genetic risk scores and longitudinal CSF biomarkers in PD patients and HCs.

		p-tau/Aβ_42_		αSyn	
Group	Predictors	β (SE)	*P*	β (SE)	*P*
PD	GRS-AD	4.25 (0.67)	**<0.001**	−0.18 (0.05)	**0.001**
	GRS-PD	0.39 (0.62)	0.538	−0.07 (0.05)	0.137
	time	0.28 (0.11)	**0.008**	−0.03 (0.01)	**0.002**
	GRS-AD*GRS-PD			0.20 (0.07)	**0.005**
	GRS-AD*time	0.27 (0.15)	0.082	0.04 (0.02)	**0.008**
	GRS-PD*time	0.29 (0.13)	**0.031**	0.02 (0.01)	0.144
	GRS-AD*GRS-PD*time			−0.05 (0.02)	**0.021**
	Time-varying αSyn	0.65 (0.35)	0.062		
	Time-varying p-tau/Aβ_42_			0.01 (0.002)	**0.010**
HC	GRS-AD	8.53 (1.84)	**<0.001**	−0.10 (0.08)	0.226
	GRS-PD	1.22 (2.08)	0.557	−0.01 (0.09)	0.886
	time	0.42 (0.26)	0.103	0.01 (0.02)	0.615
	GRS-AD*time	0.58 (0.34)	0.087	0.01 (0.02)	0.519
	GRS-PD*time	0.14 (0.36)	0.710	−0.01 (0.02)	0.835
	Time-varying αSyn	1.94 (0.72)	**0.007**		
	Time-varying p-tau/Aβ_42_			0.01 (0.002)	**<0.001**

*αSyn* Alpha-synuclein, *AD* Alzheimer’s disease, *GRS* Genetic risk score, *HC* Healthy control, *PD* Parkinson’s disease, *p-tau/Aβ*_*42*_ Phosphorylated tau/42-residue amyloid-beta.

Data are presented as the results of linear mixed models for each longitudinal CSF biomarker using GRS-AD, GRS-PD, time, GRS-AD*time, and GRS-PD*time as predictors. Significant interactions between the predictors were included as predictors. Covariates included age, sex, education, remaining CSF biomarker, and the first four principal components.

Significant *P* values are indicated in bold.

In HC subjects, LMM for time-varying CSF p-tau/Aβ showed that GRS-AD and time-varying αSyn were significantly associated with time-varying CSF p-tau/Aβ, suggesting that higher GRS-AD was associated with higher CSF p-tau/Aβ and time-varying CSF αSyn positively correlates with time-varying CSF p-tau/Aβ. LMM for time-varying CSF αSyn showed that time-varying CSF p-tau/Aβ was significantly associated with time-varying CSF αSyn, but the effects of other predictors were not significant.

### Association between GRS and longitudinal cognitive scores in PD patients

In PD patients, model 1 LMM for longitudinal cognitive scores showed that GRS-AD and GRS-AD*GRS-PD were significantly associated with visuospatial score; no predictors were associated with memory score; GRS-PD was associated with semantic fluency score; and time and GRS-PD*time were associated with frontal/executive score (Table 4). Model 2 LMM showed that the effects of GRS-AD and GRS-AD*GRS-PD were still significant for visuospatial score, although the effect of CSF biomarkers were not significant; that higher baseline CSF p-tau/Aβ*time was associated with a lower memory score; that lower GRS-PD and higher baseline CSF p-tau/Aβ*time were associated with lower semantic fluency score; and that lower GRS-PD*time and higher baseline CSF p-tau/Aβ*time were associated with lower frontal/executive score, whereas time and GRS-AD*time were not.

**Table 4:** Effects of genetic risk scores and CSF biomarkers on longitudinal cognitive changes in PD patients.

		Visuospatial		Memory		Semantic fluency		Frontal/executive	
	Predictors	β (SE)	*P*	β (SE)	*P*	β (SE)	*P*	β (SE)	*P*
Model 1	GRS-AD	−0.46 (0.14)	0.001^*^	−0.20 (0.16)	0.210	−0.12 (0.74)	0.877	−0.25 (0.59)	0.679
	GRS-PD	0.12 (0.12)	0.344	0.10 (0.15)	0.516	2.14 (0.70)	0.002^*^	0.79 (0.55)	0.150
	GRS-AD*GRS-PD	0.57 (0.17)	0.001^*^						
	time	−0.05 (0.02)	**0.033**	−0.03 (0.03)	0.284	−0.26 (0.11)	**0.018**	−0.60 (0.11)	<0.001^*^
	GRS-AD*time	−0.07 (0.03)	0.037	−0.03 (0.04)	0.532	−0.25 (0.15)	0.111	−0.37 (0.15)	0.015
	GRS-PD*time	0.03 (0.03)	0.376	0.05 (0.04)	0.214	0.07 (0.14)	0.611	0.43 (0.14)	0.002^*^
Model 2	GRS-AD	−0.50 (0.15)	0.001^*^	−0.20 (0.18)	0.268	0.04 (0.84)	0.965	−0.20 (0.65)	0.755
	GRS-PD	0.11 (0.13)	0.417	0.24 (0.16)	0.132	2.47 (0.76)	0.001^*^	0.96 (0.58)	0.100
	GRS-AD*GRS-PD	0.52 (0.18)	**0.004**						
	time	−0.0004 (0.07)	0.996	0.23 (0.09)	**0.009**	0.54 (0.32)	0.098	0.23 (0.32)	0.476
	GRS-AD*time	−0.05 (0.04)	0.155	0.04 (0.05)	0.383	−0.09 (0.16)	0.592	−0.15 (0.16)	0.357
	GRS-PD*time	0.03 (0.03)	0.287	0.05 (0.04)	0.257	−0.009 (0.146)	0.948	0.47 (0.15)	0.001^*^
	Baseline p-tau/Aβ_42_	−0.004 (0.01)	0.694	0.003 (0.01)	0.787	−0.07 (0.06)	0.245	−0.02 (0.05)	0.612
	Baseline αSyn	−0.05 (0.14)	0.743	0.09 (0.18)	0.609	0.24 (0.84)	0.776	1.14 (0.64)	0.074
	Baseline p-tau/Aβ_42_*time	−0.005 (0.003)	0.082	−0.02 (0.003)	<0.001^*^	−0.05 (0.01)	<0.001^*^	−0.07 (0.01)	<0.001^*^
	Baseline αSyn*time	0.01 (0.04)	0.700	0.02 (0.04)	0.599	0.04 (0.16)	0.799	0.18 (0.16)	0.274

*AD* Alzheimer’s disease, *GRS* Genetic risk score, *PD* Parkinson’s disease, *p-tau/Aβ*_*42*_ Phosphorylated tau/42-residue amyloid-beta.

Data are presented as the results of linear mixed models for each cognitive score, using age, sex, education, and the first four principal components as covariates. Predictors included GRS-AD, GRS-PD, GRS-AD*time, and GRS-PD*time for model 1. Baseline CSF p-tau/Aβ_42_, αSyn, p-tau/Aβ_42_*time, and αSyn*time were included as predictors in model 2.

Significant *P* values after false discovery rate correction for multiple statistical tests are indicated in bold.

### Association of GRS with longitudinal motor severity scores

In PD patients, model 1 LMM for longitudinal motor severity scores showed that the effects of time and GRS-PD*time on motor severity scores were significant, suggesting that motor severity scores increased over time and that higher GRS-PD was associated with slower aggravation in motor severity scores (Table 5). Model 2 LMM showed that the effects of time and GRS-PD on motor severity scores were significant, but that the effect of GRS-PD*time was no longer significant although the effects of time-varying CSF biomarkers were not significant. Model 3 LMM showed that the effects of time and time-varying DAT-putamen were significant, but that GRS-PD*time was no longer associated with motor severity scores. Finally, model 4 LMM, simultaneously including time-varying CSF biomarkers and time-varying DAT-putamen as predictors from model 1 LMM, showed that the effects of GRS-PD, time, and time-varying DAT-putamen were significant. These results suggested that motor severity scores increased over time; that higher GRS-PD was associated with lower motor severity scores given the same levels of time-varying CSF biomarkers and the same levels of CSF biomarkers and DAT-putamen.

**Table 5:** Effects of genetic risk scores and CSF biomarkers on longitudinal motor severity scores in PD patients.

	Model 1		Model 2		Model 3		Model 4	
Predictors	β (SE)	*P*	β (SE)	*P*	β (SE)	*P*	β (SE)	*P*
GRS-AD	0.63 (0.67)	0.347	0.35 (0.73)	0.635	0.64 (0.64)	0.318	0.21 (0.70)	0.768
GRS-PD	−1.07 (0.62)	0.088	−1.31 (0.65)	**0.046**	−1.04 (0.59)	0.080	−1.38 (0.63)	**0.028**
time	2.53 (0.15)	**<0.001**	2.73 (0.27)	**<0.001**	2.32 (0.21)	**<0.001**	2.56 (0.35)	**<0.001**
GRS-AD*time	0.18 (0.21)	0.374	0.33 (0.38)	0.384	0.03 (0.26)	0.895	0.81 (0.47)	0.087
GRS-PD*time	−0.37 (0.19)	**0.048**	−0.34 (0.34)	0.320	−0.46 (0.24)	0.059	−0.52 (0.43)	0.232
Time-varying p-tau/Aβ_42_			−0.02 (0.04)	0.626			−0.01 (0.04)	0.780
Time-varying αSyn			−0.67 (0.58)	0.243			−0.36 (0.58)	0.529
Time-varying DAT-putamen					−6.49 (1.19)	**<0.001**	−6.84 (1.32)	**<0.001**

*αSyn* Alpha-synuclein, *AD* Alzheimer’s disease, *DAT-putamen* Putaminal dopamine transporter uptake, *GRS* Genetic risk score, *PD* Parkinson’s disease, *p-tau/Aβ*_*42*_ Phosphorylated tau/42-residue amyloid-beta.

Data are presented as the results of linear mixed models for longitudinal motor severity scores, using age, sex, education, and the first four principal components as covariates. Time, GRS-AD, GRS-PD, GRS-AD*time, and GRS-PD*time were used as predictors in model 1. Time-varying CSF biomarkers were included as predictors in model 2. Time-varying DAT-putamen was included as a predictor in model 3. Model 4 included both time-varying CSF biomarkers and time-varying DAT-putamen as predictors.

Significant *P* values are indicated in bold.

### Sensitivity analyses

Fourteen of 413 PD patients (3.4%) had glucocerebrosidase (*GBA*) mutation. When the effects of *GBA* mutation on longitudinal cognitive scores were investigated, model 1 LMM showed that the presence of *GBA* mutation was associated with a faster decline in semantic fluency score (Supplementary Table 1). However, model 2 LMM showed that *GBA* mutation was no longer associated with longitudinal cognitive function scores after false discovery rate (FDR) correction. When the effects of *GBA* mutation on longitudinal motor severity scores were investigated, the presence of *GBA* mutation was associated with faster aggravation in motor severity scores given the same levels of time-varying CSF biomarkers (Supplementary Table 2).

When the effects of recalculated GRS-AD after excluding the *APOE* region on longitudinal CSF biomarkers were investigated, the effects were the same as those of the original GRS-AD, except that the recalculated GRS-AD was no longer associated with CSF αSyn levels in PD patients and CSF p-tau/Aβ_42_ levels in HC subjects (Supplementary Table 3). When the effects of recalculated GRS-AD on longitudinal cognitive scores were investigated, the results were the same as the original results, except that the recalculated GRS-AD was no longer associated with semantic fluency score in model 1 LMM (Supplementary Table 4).

## Discussion

In this study, we evaluated the relationship between GRS-AD and GRS-PD with longitudinal CSF biomarkers and clinical outcomes in de novo PD patients. Our major findings are as follows. First, higher GRS-PD and lower CSF αSyn were independently associated with an increased risk of PD. Second, higher GRS-AD was associated with a higher level of CSF p-tau/Aβ and a lower level of CSF αSyn, while it was associated with a faster decrease in CSF αSyn. Third, higher baseline CSF p-tau/Aβ and GRS-AD were associated with cognitive decline in PD patients. Fourth, higher GRS-PD was associated with better semantic fluency domain, slower decline in frontal/executive function, and better motor symptoms, given the same level of CSF biomarkers. Our results suggest that higher GRS-AD and CSF biomarkers for AD are associated with cognitive declines in PD, while higher GRS-PD represents an increased risk of PD, albeit slower clinical progression after PD diagnosis.

Higher GRS-PD and lower baseline CSF αSyn levels were independently associated with an increased risk of PD. Previous studies have shown that CSF αSyn is decreased at baseline in PD^7^ and that GRS-PD is associated with diagnosis^21^ and younger symptom onset of PD^14,15^. However, another study showed that lower CSF Aβ and p-tau were associated with PD diagnosis, rather than lower CSF αSyn^6^. This discrepancy is probably due to the high correlation between CSF αSyn and AD-related CSF biomarkers. Neuronal damage caused by AD pathology could lead to the release of neuronal tau and α-syn into the CSF, resulting in increases therein in CSF^8,9^. Supporting this point of view, a previous study proposed that mismatch of high CSF p-tau and low CSF αSyn indicates the presence of additional LB pathology in AD patients^8^. Thus, our approach that simultaneously considers CSF AD biomarkers and CSF α-syn, along with GRS-AD and GRS-PD, may have enabled us to find a significant association between CSF αSyn and PD.

Higher GRS-AD was associated with higher CSF p-tau/Aβ and lower CSF αSyn levels. The direction of β coefficients for time in Table 3 indicated that CSF p-tau/Aβ increased and CSF αSyn decreased in PD patients over time. These results suggest that AD- and LBD-related pathologies could progress, and that AD-related pathophysiology could aggravate LBD-related pathology in de novo PD patients. Supporting this point of view, previous autopsy studies have shown that LBD patients with AD co-pathology exhibit greater neocortical αSyn pathology^22,23^. However, the direction of GRS-AD effects on longitudinal change of CSF αSyn (GRS-AD*time) was reversed, such that higher GRS-AD was associated with slower decrease in CSF αSyn. A possible explanation for this complex relationship between GRS-AD and CSF αSyn could be that higher GRS-AD may not only aggravate αSyn pathology but also induce faster neuronal damage leading to relative increases in CSF αSyn level.

Higher baseline CSF p-tau/Aβ was associated with a faster decline in memory, semantic fluency, and frontal/executive function in PD patients. Although a previous study in the PPMI cohort found an association between CSF Aβ and working memory, rather than episodic memory^10^, the effects of CSF αSyn were not simultaneously considered in the study due to high correlation between CSF biomarkers^24^. In our study, we used CSF p-tau/Aβ to avoid issues with multi-collinearity. Considering that concomitant AD co-pathology in LBD patients is reflected in CSF biomarkers, including tau and Aβ^2^, our results are consistent with previous studies showing a strong influence of AD pathology on cognitive dysfunction in PD patients^4,23,25,26^. Thus, our results suggest that CSF p-tau/Aβ could be a biomarker for AD-related pathophysiology in PD patients when CSF αSyn is simultaneously considered. It is noteworthy that higher GRS-AD was associated with visuospatial dysfunction, but CSF p-tau/Aβ was not. Considering that visuospatial dysfunction is a cognitive characteristic of DLB^24^ and a mixed disease of AD with LBD^27,28^, unknown LB-related mechanisms, such as the interaction of Aβ and LB-related brain changes converging in the occipital cortex^28^, could explain visuospatial dysfunction related to GRS-AD.

Higher GRS-PD was associated with better semantic fluency domain, slower decline in frontal/executive function, and better motor symptoms, given the same level of CSF biomarkers. These findings are not consistent with previous studies showing faster cognitive decline and motor progression in PD patients with higher GRS-PD^16,17^. The discrepancy could be attributable to the different set of SNPs used for GRS, demographic differences of the cohorts, and further consideration of CSF biomarkers and AD-GRS in our study. To evaluate the validity of the model used in our study, we conducted sensitivity analyses using the presence of *GBA* mutation as a predictor instead of PD-GRS (Supplementary Tables 1 and 2). Although the number of *GBA* mutation carriers was small (*n* = 14; 3.4%), those with *GBA*-associated PD showed faster cognitive declines in semantic fluency scores and faster motor progression, consistent with previous studies^29,30^. There is a PPMI-based study that showed slower striatal dopaminergic degeneration and motor outcomes in those with higher GRS-PD, possibly due to the heterogenetic effects of alleles used for GRS on the progression of PD^18^. Considering that higher GRS-PD was associated with the risk of PD, PD patients with higher GRS-PD may be more susceptible to the clinical symptoms of PD with the same burden of αSyn pathology rather than more rapid spreading of αSyn pathology. As higher GRS-PD was associated with slower cognitive and motor progression given the same CSF biomarker burden after disease onset, the effect of higher GRS-PD in PD patients resembled the effect of lower education in AD patients^31^. As a proxy marker for cognitive reserve, lower education is associated with a higher risk of developing AD dementia given the same amount of AD pathology, but it is related to slower symptom progression after disease onset. Relative genetic composition of GRS-PD, either related to the burden of αSyn pathology or functional reserve against αSyn pathology, could explain the discrepancy in the previously reported effect of GRS-PD on clinical progression, either protective^15,18^ or harmful^16,17^. Inconsistency on the effects of GRS-PD requires further application of GRS-PD on multiple cohorts and large-sized meta-analyses.

Our study had several limitations. First, a direct mechanism for the association between individual risk loci consisting of GRS and cognitive impairment or CSF biomarkers needs to be studied. Second, as the follow-up period of our analyses is relatively short, extrapolation of the effects of CSF biomarkers or GRS to longer periods or end-stage PD needs careful consideration. Third, a SNP within the *APOE* region was included in the computation of GRS-AD and limits the interpretation of GRS-AD other than *APOE4*, which are known to increase the risk of cognitive decline in PD^5,32,33^. In the sensitivity analyses using GRS-AD excluding a SNP in *APOE* region (Supplementary Tables 3 and 4), the estimated effect sizes and directions on CSF p-tau/Aβ and visuospatial score did not change meaningfully, suggesting that GRS-AD other than the *APOE* region may also contribute to CSF AD biomarkers and visuospatial dysfunction in PD patients.

Taken together, our data suggest that higher GRS-AD and higher CSF p-tau/Aβ reflect AD-related pathophysiology in PD, while higher GRS-PD and lower CSF αSyn could reflect PD-related pathophysiology leading to the occurrence of PD. There may be a complex interplay between AD- and PD-related pathophysiology, which supports the view that mixed AD pathology needs to be considered in PD patients.

## Methods

### Study participants

The data used in this study were obtained from the PPMI database (www.ppmi-info.org/data), which is an observational multicenter study with clinical, imaging, and biological data to identify biomarkers of PD progression. The analyses of this study are based on the initial phase of the PPMI, which enrolled 423 de novo PD participants without rare genetic mutations. We further selected 413 PD patients and 195 HCs with available whole-genome sequencing data. All procedures in the study involving human participants were performed in accordance with the ethical standards committee at each participating institution, and written informed consent was obtained from all participants. The study was registered at clinicaltrials.gov (NCT01141023).

### Genotyping and Calculation of GRS

DNA samples were extracted from whole blood and sequenced using an Illumina HiSeq X Ten Sequencer. Paired-end reads were aligned to the reference genome, and single nucleotide polymorphisms (SNPs) were called using the Genome Analysis Tool Kit^34^. We extracted the genotypes of the AD- and PD-associated risk loci^21,35^, with the following quality control criteria: SNPs on non-autosomes, multi-allelic, low minor allele frequencies (<0.005), deviation from Hardy–Weinberg equilibrium (*P* < 1E-6), and high rates of missing genotypes (>0.05). GRS-AD was computed using genotypes of 24 risk loci for AD (Supplementary Table 5)^35^, while GRS-PD was computed using genotypes of 87 risk loci for PD (Supplementary Table 6)^21^. Three risk loci for PD were excluded as the variants were multiallelic. As GRS-PD including those SNPs was highly correlated with those without (*r* = 99.5; *p* < 0.001), we conducted the analyses with GRS-PD based on the 87 bi-allelic risk loci. Each GRS was calculated by summing the number of risk alleles weighted by log-transformed odds ratios (OR). To account for genomic population structures, we conducted a principal component (PC) analysis using each participant’s SNP data using PLINK^36^. The first four PCs were included as covariates in all statistical analyses. To identify the effects of SNPs other than *APOE* on cognitive scores, we computed GRS-AD without the SNP rs429358 on the *APOE* region. To evaluate the effects of mutations in *GBA*, which is known to affect cognition in PD patients, the presence of *GBA* mutation was investigated including 84GG, N370S, L444P, A456P, and IVS2 + 1 G > A.

### Clinical assessment and CSF biomarkers

Baseline and longitudinal scores for cognitive domains were measured as follows: (i) memory, delayed recall score of Hopkins Verbal Learning Test Revised; (ii) semantic fluency domain; (iii) frontal/executive, Symbol Digit Modality score, and (iv) visuospatial function, Benton Judgment of Line Orientation score. For motor severity score, we used the Movement Disorders Society modified Unified Parkinson’s Disease Rating Scale (MDS-UPDRS) part III total score.

Baseline and longitudinal (up to 3 years from baseline) CSF biomarkers, including Aβ, t-tau, and p-tau, were measured using Elecsys immunoassays on the Cobas e 601 platform (*Roche Diagnostics*). The range of quantitation for CSF Aβ is from 0.2 ng/mL to 1.7 ng/mL^37^, and the extrapolated value for CSF Aβ over 1.7 ng/mL was used. The cutoff for CSF amyloid-positivity was set to 1.1 ng/mL^38^. CSF αSyn was measured using ELISA (BioLegend).

### Dopamine transporter imaging

All participants underwent DAT scans using ^123^I-FP-CIT following the PPMI imaging protocol. Processed data were normalized to the standard Montreal Neurologic Institute space, and the occipital cortex was used as a reference for quantitative analysis. The mean specific binding ratio, which is calculated as (target region/reference region)-1, for the bilateral putamen was selected for analyses using the DAT imaging biomarker.

### Statistical analysis

Statistical analyses of the demographic and clinical data were performed using IBM SPSS version 26.0 (SPSS Inc., Chicago, IL, USA) and R Statistical Software (version 4.1.0; R Foundation for Statistical Computing, Vienna, Austria). Analysis of variance and chi-square test were used to compare demographic variables between PD patients and HCs. Due to strong correlations between CSF tau, p-tau, and αSyn, CSF p-tau/Aβ and CSF αSyn were selected as predictors to avoid multicollinearity issues in the following analyses. All analyses included age, sex, education, and the first four PCs as covariates. A logistic regression model was used to evaluate ORs for the diagnosis of PD using either GRS-AD, GRS-PD (model 1), CSF biomarkers (model 2), or both (model 3) as predictors. To evaluate the performance of GRS and CSF biomarkers in the classification of LBD patients from HCs, ROC curve analyses were performed in all participants using GRS-AD and GRS-PD (Model 1), CSF biomarkers (Model 2), and GRS-AD, GRS-PD, and CSF biomarkers (Model 3). The optimal cutoff points on the ROC curves were determined using the Youden index, and the area under the curve of each model was compared to that of Model 2 using DeLong’s test.

To evaluate the association between longitudinal effects of GRSs on clinical outcomes and CSF and imaging biomarkers, we conducted LMMs with a random slope and a random intercept using the *lme4* package of R. Follow-up years from baseline were used for time predictors in the model. Interaction terms between GRSs and time (year) were used as predictors to measure the effect of GRS on the rate of changes in outcome variables. To evaluate the association between longitudinal CSF biomarkers and GRS, LMMs for time-varying CSF biomarkers were performed using GRS-AD, GRS-PD, time (in years), GRS-AD*time, and GRS-PD*time as predictors in PD patients and HCs separately. Significant interactions between the predictors and the remaining CSF biomarkers were further included as predictors. The effects of GRS-AD and GRS-PD on longitudinal cognitive scores were evaluated using LMMs in PD patients. Predictors included GRS, time, GRS*time, and significant interactions between the predictors (model 1). To evaluate the independent effects of GRS and baseline CSF biomarkers on longitudinal cognitive scores, baseline CSF p-tau/Aβ, baseline CSF αSyn, and their interactions with time were added to the predictors from model 1 (model 2). As we performed LMMs for four cognitive scores, the FDR method was used to correct for multiple comparisons.

To evaluate the longitudinal effects of GRS on motor severity scores in PD patients, LMMs for time-varying motor severity scores were performed using GRS, time, and GRS*time as predictors (model 1). To evaluate the effects of GRS given the same level of either CSF biomarkers or DAT-putamen, time-varying CSF biomarkers (model 2), time-varying DAT-putamen (model 3), or both (model 4) were included as predictors.

To determine the effects of *GBA* mutation on longitudinal cognitive scores and motor severity scores, sensitivity analyses were performed using the presence of *GBA* mutation as a predictor instead of GRS-PD. Also, to evaluate the effects of GRS-AD other than *APOE*, additional sensitivity analyses using the recalculated GRS-AD excluding the rs429358 on *APOE* region were performed.

### Reporting summary

Further information on research design is available in the Nature Research Reporting Summary linked to this article.

## Supplementary information

Supplementary_materials

Reporting Summary Checklist

## Data Availability

All data generated or analyzed during this study are available from the PPMI website (https://www.ppmi-info.org/access-data-specimens/download-data/).

## Code availability

R code for the statistical analyses of this study is available at https://github.com/ygunlee/PPMI_PRS_CSF.
