# Supplementary material for: Effects of Alzheimer’s genetic risk scores and CSF biomarkers in de novo Parkinson’s Disease
Source: NPJ Parkinsons Dis. 2022 May 11;8:57. doi: 10.1038/s41531-022-00317-8 (PMC9095668; doi:10.1038/s41531-022-00317-8)
Supplement: Supplementary file 1 — Supplementary_materials [file 41531_2022_317_MOESM1_ESM.docx]

**Supplementary Table 1. Effect of *GBA* mutations, GRS-AD, and CSF biomarkers on longitudinal cognitive changes in PD patients**

|  |  | Visuospatial | | Memory | | Semantic fluency | | Frontal/executive | |
| --- | --- | --- | --- | --- | --- | --- | --- | --- | --- |
|  | Predictors | β (SE) | *P* | β (SE) | *P* | β (SE) | *P* | β (SE) | *P* |
| Model 1 | GRS-AD | -0.31 (0.13) | 0.018 | -0.21 (0.16) | 0.197 | -0.16 (0.76) | 0.829 | -0.22 (0.59) | 0.709 |
|  | *GBA* | 0.07 (0.48) | 0.876 | 0.490 (0.583) | 0.401 | 5.43 (2.74) | 0.048 | -2.15 (2.15) | 0.318 |
|  | time | -0.04 (0.02) | 0.054 | -0.01 (0.03) | 0.673 | -0.20 (0.11) | 0.060 | -0.52 (0.11) | **< 0.001^*^** |
|  | GRS-AD*time | -0.07 (0.03) | 0.040 | -0.02 (0.04) | 0.611 | -0.22 (0.15) | 0.147 | -0.38 (0.15) | 0.014 |
|  | *GBA**time | -0.01 (0.11) | 0.914 | -0.26 (0.15) | 0.086 | -1.43 (0.54) | **0.008** | 0.81 (0.54) | 0.137 |
| Model 2 | GRS-AD | -0.35 (0.15) | 0.015 | -0.21 (0.18) | 0.242 | -0.04 (0.86) | 0.964 | -0.20 (0.65) | 0.759 |
|  | *GBA* | 0.47 (0.49) | 0.339 | 0.72 (0.61) | 0.245 | 6.05 (2.93) | 0.040 | -0.80 (2.21) | 0.717 |
|  | time | -0.01 (0.07) | 0.846 | 0.26 (0.09) | **0.004** | 0.59 (0.32) | 0.065 | 0.33 (0.32) | 0.303 |
|  | GRS-AD*time | -0.05 (0.04) | 0.179 | 0.05 (0.05) | 0.301 | -0.06 (0.16) | 0.702 | -0.15 (0.17) | 0.362 |
|  | *GBA**time | -0.02 (0.01) | 0.881 | -0.02 (0.15) | 0.111 | -1.21 (0.55) | 0.028 | 1.01 (0.55) | 0.068 |
|  | Baseline p-tau/Aβ_42_ | -0.01 (0.01) | 0.594 | 0.004 (0.013) | 0.746 | -0.07 (0.06) | 0.286 | -0.02 (0.05) | 0.664 |
|  | Baseline αSyn | -0.01 (0.01) | 0.918 | 0.08 (0.18) | 0.654 | 0.14 (0.84) | 0.866 | 1.07 (0.64) | 0.094 |
|  | Baseline p-tau/Aβ_42_*time | -0.004 (0.003) | 0.071 | -0.02 (0.003) | **< 0.001^*^** | -0.05 (0.01) | **< 0.001^*^** | -0.07 (0.01) | **< 0.001^*^** |
|  | Baseline αSyn*time | 0.01 (0.04) | 0.727 | 0.02 (0.04) | 0.628 | 0.04 (0.16) | 0.817 | 0.17 (0.16) | 0.307 |

Abbreviations: AD: Alzheimer’s disease, GRS: Genetic risk score, PD: Parkinson’s disease, p-tau/Aβ_42_: Phosphorylated tau/42-residue amyloid-beta.

Data are presented as the results of linear mixed models for each cognitive score, using age, sex, education, and the first four principal components as covariates. Predictors included GRS-AD, GRS-PD, GRS-AD*time, and GRS-PD*time in model 1. Baseline CSF p-tau/Aβ_42_, αSyn, p-tau/Aβ_42_*time, and αSyn*time were included as predictors in model 2.

Significant *P* values after applying the false discovery rate method for multiple statistical tests are indicated in bold.

**Supplementary Table 2. Effect of *GBA* mutations, GRS-AD, and CSF biomarkers on longitudinal motor severity scores in PD patients**

|  | Model 1 |  | Model 2 |  | Model 3 |  | Model 4 |  |
| --- | --- | --- | --- | --- | --- | --- | --- | --- |
| Predictors | β (SE) | *P* | β (SE) | *P* | β (SE) | *P* | β (SE) | *P* |
| GRS-AD | 0.63 (0.67) | 0.350 | 0.42 (0.73) | 0.564 | 0.65 (0.64) | 0.311 | 0.28 (0.70) | 0.693 |
| *GBA* | 0.58 (2.43) | 0.813 | -2.26 (2.47) | 0.361 | -0.39 (2.30) | 0.867 | -4.36 (2.41) | 0.072 |
| time | 2.45 (0.15) | **< 0.001** | 2.53 (0.26) | **< 0.001** | 2.21 (0.20) | **< 0.001** | 2.30 (0.34) | **< 0.001** |
| GRS-AD*time | 0.17 (0.21) | 0.403 | 0.22 (0.38) | 0.571 | 00.01 (0.26) | 0.965 | 0.71 (0.47) | 0.131 |
| *GBA**time | -0.32 (0.78) | 0.683 | 4.01 (1.36) | **0.004** | 0.08 (0.99) | 0.933 | 3.91 (1.60) | **0.015** |
| Time-varying p-tau/Aβ_42_ |  |  | -0.03 (0.05) | 0.534 |  |  | -0.02 (0.04) | 0.695 |
| Time-varying αSyn |  |  | -0.63 (0.58) | 0.273 |  |  | -0.32 (0.58) | 0.582 |
| Time-varying DAT-putamen |  |  |  |  | -6.44 (1.19) | **< 0.001** | -6.78 (1.32) | **< 0.001** |

Abbreviations: αSyn: Alpha-synuclein, AD: Alzheimer’s disease, DAT-putamen: Putaminal dopamine transporter uptake, GRS: Genetic risk score, PD: Parkinson’s disease, p-tau/Aβ_42_: Phosphorylated tau/42-residue amyloid-beta.

Data are presented as the results of linear mixed models for longitudinal motor severity scores, using age, sex, education, and the first four principal components as covariates. Time, GRS-AD, GRS-PD, GRS-AD*time, and GRS-PD*time were used as predictors in model 1. Time-varying CSF biomarkers were included as predictors in model 2. Time-varying DAT-putamen was included as a predictor in model 3. Model 4 included both time-varying CSF biomarkers and time-varying DAT-putamen as predictors.

Significant *P* values are indicated in bold.

**Supplementary Table 3. Association between genetic risk scores excluding rs429358** **and longitudinal CSF biomarkers in PD patients and HCs**

|  |  | p-tau/Aβ_42_ |  | αSyn |  |
| --- | --- | --- | --- | --- | --- |
| Group | Predictors | β (SE) | *P* | β (SE) | *P* |
| PD | GRS-AD^a^ | 4.46 (1.46) | **0.002** | -0.16 (0.11) | 0.122 |
|  | GRS-PD | 0.25 (0.66) | 0.702 | 0.004 (0.047) | 0.932 |
|  | time | 0.39 (0.11) | **0.001** | -0.02 (0.01) | 0.122 |
|  | GRS-AD^a^*GRS-PD |  |  | 0.44 (0.14) | **0.002** |
|  | GRS-AD^a^*time | 0.46 (0.31) | 0.138 | 0.06 (0.03) | **0.036** |
|  | GRS-PD*time | 0.28 (0.13) | **0.035** | 0.004 (0.013) | 0.769 |
|  | GRS-AD^a^*GRS-PD*time |  |  | -0.09 (0.04) | **0.012** |
|  | Time-varying αSyn | 0.55 (0.35) | 0.115 |  |  |
|  | Time-varying p-tau/Aβ_42_ |  |  | 0.004 (0.002) | **0.040** |
| HC | GRS-AD^a^ | 4.00 (4.38) | 0.362 | 0.01 (0.17) | 0.977 |
|  | GRS-PD | 0.63 (2.20) | 0.773 | -0.01 (0.09) | 0.931 |
|  | time | 0.70 (0.27) | **0.011** | 0.004 (0.018) | 0.813 |
|  | GRS-AD^a^*time | 1.22 (0.84) | 0.151 | -0.06 (0.06) | 0.287 |
|  | GRS-PD*time | 0.09 (0.36) | 0.810 | -0.01 (0.02) | 0.811 |
|  | Time-varying αSyn | 1.91 (0.72) | **0.009** |  |  |
|  | Time-varying p-tau/Aβ_42_ |  |  | 0.01 (0.002) | **< 0.001** |

Abbreviations: αSyn: Alpha-synuclein, AD: Alzheimer’s disease, GRS: genetic risk score, HC: Healthy control, PD: Parkinson’s disease, p-tau/Aβ_42_: Phosphorylated tau/42-residue amyloid-beta.

Data are presented as the results of linear mixed models for each longitudinal CSF biomarker using GRS-AD, GRS-PD, time, GRS-AD*time, and GRS-PD*time as predictors. Significant interactions between the predictors were included as predictors. Covariates included age, sex, education, remaining CSF biomarker, and the first four principal components. A SNP in the *APOE* region was excluded in computation of GRS-AD.

Significant *P* values are indicated in bold.

^a^GRS-AD was computed after excluding rs429358.

**Supplementary Table 4. Effect of genetic risk scores excluding rs429358** **and CSF biomarkers on longitudinal cognitive changes in PD patients**

|  |  | Visuospatial | | Memory | | Semantic fluency | | Frontal/executive | |
| --- | --- | --- | --- | --- | --- | --- | --- | --- | --- |
|  | Predictors | β (SE) | *P* | β (SE) | *P* | β (SE) | *P* | β (SE) | *P* |
| Model 1 | GRS-AD^a^ | -0.67 (0.27) | **0.014** | -0.62 (0.33) | 0.063 | -3.53 (1.55) | **0.024** | -1.73 (1.23) | 0.679 |
|  | GRS-PD | 0.24 (0.12) | 0.051 | 0.11 (0.15) | 0.449 | 2.23 (0.69) | **0.001** | 0.79 (0.55) | 0.150 |
|  | time | -0.07 (0.02) | **0.006** | -0.04 (0.03) | 0.189 | -0.33 (0.11) | **0.003** | -0.60 (0.11) | **< 0.001** |
|  | GRS-AD^a^*time | -0.02 (0.07) | 0.704 | -0.02 (0.09) | 0.784 | -0.18 (0.31) | 0.560 | -0.37 (0.15) | 0.015 |
|  | GRS-PD*time | 0.02 (0.03) | 0.408 | 0.05 (0.04) | 0.215 | 0.07 (0.14) | 0.614 | 0.43 (0.14) | **0.002** |
| Model 2 | GRS-AD^a^ | -0.80 (0.29) | **0.007** | -0.76 (0.36) | 0.037 | -3.19 (1.70) | 0.061 | -1.91 (1.30) | 0.143 |
|  | GRS-PD | 0.23 (0.13) | 0.078 | 0.26 (0.16) | 0.102 | 2.57 (0.76) | **0.001** | 1.02 (0.58) | 0.080 |
|  | time | -0.01 (0.07) | 0.937 | 0.25 (0.01) | **0.005** | 0.50 (0.33) | 0.129 | 0.20 (0.33) | 0.542 |
|  | GRS-AD^a^*time | -0.02 (0.07) | 0.802 | 0.08 (0.09) | 0.377 | -0.09 (0.32) | 0.774 | -0.06 (0.33) | 0.857 |
|  | GRS-PD*time | 0.03 (0.03) | 0.333 | 0.04 (0.04) | 0.268 | -0.01 (0.15) | 0.957 | 0.47 (0.15) | **0.001** |
|  | Baseline p-tau/Aβ_42_ | -0.01 (0.01) | 0.339 | 0.003 (0.01) | 0.792 | -0.05 (0.06) | 0.357 | -0.02 (0.04) | 0.680 |
|  | Baseline αSyn | 0.03 (0.14) | 0.802 | 0.11 (0.18) | 0.521 | 0.20 (0.83) | 0.806 | 1.15 (0.63) | 0.069 |
|  | Baseline p-tau/Aβ_42_*time | -0.01 (0.003) | 0.038 | -0.02 (0.003) | **< 0.001** | -0.05 (0.01) | **< 0.001** | -0.07 (0.01) | **< 0.001** |
|  | Baseline αSyn*time | 0.02 (0.04) | 0.588 | 0.02 (0.04) | 0.681 | 0.05 (0.16) | 0.736 | 0.20 (0.16) | 0.219 |

Abbreviations: AD: Alzheimer’s disease, GRS: Genetic risk score, PD: Parkinson’s disease, p-tau/Aβ_42_: Phosphorylated tau/42-residue amyloid-beta.

Data are presented as the results of linear mixed models for each cognitive score, using age, sex, and education as covariates. Predictors included GRS-AD, GRS-PD, GRS-AD*time, and GRS-PD*time for model 1. Baseline CSF p-tau/Aβ_42_, αSyn, p-tau/Aβ_42_*time, and αSyn*time were included as predictors for model 2. A SNP in *APOE* region was excluded in computation of GRS-AD.

Significant *P* values after false discovery rate method for multiple statistical tests were in bold.

^a^GRS-AD was computed after excluding rs429358.

**Supplementary Table 5. AD-associated SNPs used for GRS-AD**

| SNP | Location (chr:locus) | Nearest gene | Effective allele | OR |
| --- | --- | --- | --- | --- |
| rs4844610 | 1:207802552 | *CR1* | A | 1.17 |
| rs6733839 | 2:127892810 | *BIN1* | T | 1.2 |
| rs10933431 | 2:233981912 | *INPP5D* | G | 0.91 |
| rs9271058 | 6:32575406 | *HLA-DRB1* | A | 1.1 |
| rs75932628 | 6:41129252 | *TREM2* | T | 2.08 |
| rs9473117 | 6:47431284 | *CD2AP* | C | 1.09 |
| rs12539172 | 7:100091795 | *NYAP1* | T | 0.92 |
| rs10808026 | 7:143099133 | *EPHA1* | A | 0.9 |
| rs73223431 | 8:27219987 | *PTK2B* | T | 1.1 |
| rs9331896 | 8:27467686 | *CLU* | C | 0.88 |
| rs7920721 | 10:11720308 | *ECHDC3* | G | 1.08 |
| rs3740688 | 11:47380340 | *SPI1* | G | 0.92 |
| rs7933202 | 11:59936926 | *MS4A2* | C | 0.89 |
| rs3851179 | 11:85868640 | *PICALM* | T | 0.88 |
| rs11218343 | 11:121435587 | *SORL1* | C | 0.8 |
| rs17125924 | 14:53391680 | *FERMT2* | G | 1.14 |
| rs12881735 | 14:92932828 | *SLC24A4* | C | 0.92 |
| rs593742 | 15:59045774 | *ADAM10* | G | 0.93 |
| rs62039712 | 16:79355857 | *WWOX* | A | 1.16 |
| rs138190086 | 17:61538148 | *ACE* | A | 1.32 |
| rs3752246 | 19:1056492 | *ABCA7* | G | 1.15 |
| rs429358 | 19:45411941 | *APOE* | C | 3.32 |
| rs6024870 | 20:54997568 | *CASS4* | A | 0.88 |
| rs2830500 | 21:28156856 | *ADAMTS1* | A | 0.93 |

**Supplementary Table 6. PD-associated SNPs used for GRS-PD**

| SNP | Location (chr:locus) | Nearest gene | Effective allele | OR |
| --- | --- | --- | --- | --- |
| rs114138760 | 1:154898185 | PMVK | C | 1.32 |
| rs35749011 | 1:155135036 | KRTCAP2 | A | 1.83 |
| rs76763715 | 1:155205634 | GBAP1 | T | 0.47 |
| rs6658353 | 1:161469054 | FCGR2A | C | 1.07 |
| rs11578699 | 1:171719769 | VAMP4 | T | 0.93 |
| rs823118 | 1:205723572 | NUCKS1 | T | 1.11 |
| rs11557080 | 1:205737739 | RAB29 | A | 1.14 |
| rs4653767 | 1:226916078 | ITPKB | T | 1.09 |
| rs10797576 | 1:232664611 | SIPA1L2 | T | 1.12 |
| rs76116224 | 2:18147848 | KCNS3 | A | 1.12 |
| rs2042477 | 2:96000943 | KCNIP3 | A | 0.94 |
| rs11683001 | 2:102396963 | MAP4K4 | A | 1.07 |
| rs57891859 | 2:135464616 | TMEM163 | A | 1.08 |
| rs1474055 | 2:169110394 | STK39 | T | 1.20 |
| rs73038319 | 3:18361759 | SATB1 | A | 0.84 |
| rs6808178 | 3:28705690 | LINC00693 | T | 1.07 |
| rs12497850 | 3:48748989 | IP6K2 | T | 1.07 |
| rs55961674 | 3:122196892 | KPNA1 | T | 1.09 |
| rs11707416 | 3:151108965 | MED12L | A | 0.94 |
| rs1450522 | 3:161077630 | SPTSSB | A | 0.94 |
| rs10513789 | 3:182760073 | MCCC1 | T | 1.16 |
| rs873786 | 4:925376 | GAK | T | 0.84 |
| rs34311866 | 4:951947 | TMEM175 | T | 0.81 |
| rs4698412 | 4:15737348 | BST1 | A | 1.11 |
| rs34025766 | 4:17968811 | LCORL | A | 0.92 |
| rs4101061 | 4:77147969 | FAM47E | A | 0.91 |
| rs6854006 | 4:77198054 | FAM47E-STBD1 | T | 0.91 |
| rs356182 | 4:90626111 | SNCA | A | 0.76 |
| rs5019538 | 4:90636630 | SNCA | A | 0.86 |
| rs13117519 | 4:114369065 | CAMK2D | T | 1.09 |
| rs62333164 | 4:170583157 | CLCN3 | A | 0.94 |
| rs1867598 | 5:60137959 | ELOVL7 | A | 0.86 |
| rs26431 | 5:102365794 | PAM | C | 1.06 |
| rs11950533 | 5:134199105 | C5orf24 | A | 0.91 |
| rs4140646 | 6:27738801 | LOC100131289 | A | 1.09 |
| rs9261484 | 6:30108683 | TRIM40 | T | 0.94 |
| rs112485576 | 6:32578772 | HLA-DRB5 | A | 0.85 |
| rs12528068 | 6:72487762 | RIMS1 | T | 1.07 |
| rs997368 | 6:112243291 | FYN | A | 1.07 |
| rs75859381 | 6:133210361 | RPS12 | T | 0.80 |
| rs199351 | 7:23300049 | GPNMB | A | 1.11 |
| rs1293298 | 8:11712443 | CTSB | A | 1.10 |
| rs620513 | 8:16697593 | FGF20 | T | 0.92 |
| rs2280104 | 8:22525980 | BIN3 | T | 1.06 |
| rs2086641 | 8:130901909 | FAM49B | T | 0.94 |
| rs13294100 | 9:17579690 | SH3GL2 | T | 0.92 |
| rs10756907 | 9:17727065 | SH3GL2 | A | 0.91 |
| rs6476434 | 9:34046391 | UBAP2 | T | 0.94 |
| rs896435 | 10:15557406 | ITGA8 | T | 1.08 |
| rs10748818 | 10:104015279 | GBF1 | A | 0.92 |
| rs72840788 | 10:121415685 | BAG3 | A | 1.08 |
| rs117896735 | 10:121536327 | INPP5F | A | 1.55 |
| rs7938782 | 11:10558777 | RNF141 | A | 1.09 |
| rs12283611 | 11:83487277 | DLG2 | A | 0.94 |
| rs3802920 | 11:133787001 | IGSF9B | T | 1.11 |
| rs76904798 | 12:40614434 | LRRK2 | T | 1.15 |
| rs34637584 | 12:40734202 | LRRK2 | A | 11.35 |
| rs7134559 | 12:46419086 | SCAF11 | T | 0.95 |
| rs10847864 | 12:123326598 | HIP1R | T | 1.16 |
| rs11610045 | 12:133063768 | FBRSL1 | A | 1.06 |
| rs9568188 | 13:49927732 | CAB39L | T | 1.06 |
| rs4771268 | 13:97865021 | MBNL2 | T | 1.07 |
| rs12147950 | 14:37989270 | MIPOL1 | T | 0.95 |
| rs11158026 | 14:55348869 | GCH1 | T | 0.92 |
| rs3742785 | 14:75373034 | RPS6KL1 | A | 1.07 |
| rs979812 | 14:88464264 | GALC | T | 1.06 |
| rs2251086 | 15:61997385 | VPS13C | T | 0.89 |
| rs6497339 | 16:19277493 | SYT17 | A | 1.07 |
| rs2904880 | 16:28944396 | CD19 | C | 0.94 |
| rs11150601 | 16:30977799 | SETD1A | A | 1.09 |
| rs6500328 | 16:50736656 | NOD2 | A | 1.06 |
| rs3104783 | 16:52636242 | CASC16 | A | 1.07 |
| rs10221156 | 16:52969426 | CHD9 | A | 0.89 |
| rs12600861 | 17:7355621 | CHRNB1 | A | 0.95 |
| rs12951632 | 17:40741013 | RETREG3 | T | 1.07 |
| rs2269906 | 17:42294337 | UBTF | A | 1.07 |
| rs850738 | 17:42434630 | FAM171A2 | A | 0.93 |
| rs62053943 | 17:43744203 | CRHR1 | T | 0.76 |
| rs117615688 | 17:43798308 | CRHR1 | A | 0.79 |
| rs11658976 | 17:44866805 | WNT3 | A | 0.94 |
| rs61169879 | 17:59917366 | BRIP1 | T | 1.09 |
| rs666463 | 17:76425480 | DNAH17 | A | 1.08 |
| rs1941685 | 18:31304318 | ASXL3 | T | 1.05 |
| rs12456492 | 18:40673380 | RIT2 | A | 0.91 |
| rs55818311 | 19:2341047 | SPPL2B | T | 0.93 |
| rs77351827 | 20:6006041 | CRLS1 | T | 1.08 |
| rs2248244 | 21:38852361 | DYRK1A | A | 1.07 |
